# Analysis of etiology and clinical features of spontaneous downbeat nystagmus: a retrospective study

**DOI:** 10.3389/fneur.2024.1326879

**Published:** 2024-02-01

**Authors:** Sai Zhang, Yilin Lang, Wenting Wang, Yuexia Wu, Shuangmei Yan, Ting Zhang, Dong Li, Shaona Liu, Yongci Hao, Xu Yang, Ping Gu

**Affiliations:** ^1^Department of Neurology, The First Hospital of Hebei Medical University, Shijiazhuang, Hebei, China; ^2^Vertigo Center of the First Hospital of Hebei Medical University, Shijiazhuang, Hebei, China; ^3^Department of Neurology, Peking University Aerospace School of Clinical Medicine, Beijing, China

**Keywords:** vestibular, downbeat nystagmus, cerebellar, vestibular migraine, dizziness, vertigo

## Abstract

**Objective:**

To investigate the topical diagnosis, possible etiology and mechanism of spontaneous downbeat nystagmus (sDBN) patients with dizziness/vertigo.

**Methods:**

The clinical features of dizziness/vertigo patients accompanied with DBN were retrospectively reviewed in the Vertigo Center of our hospital from January 2018 to March 2021. The clinical features of dizziness/vertigo patients accompanied with DBN were reviewed. Comprehensive VNG, bithermal caloric testing, video-head-impulse test (vHIT), vestibular-evoked myogenic potentials (VEMP), head magnetic resonance imaging (MRI), three-dimensional fluid-attenuated incersion recovery magnetic resonance imaging (3D-FLAIR MRI) in the inner ear, serum immunology and other examinations were to determine the lesion site, and analyze its possible etiology and mechanism.

**Results:**

A total of 54 patients were included. Among them, 70.4% (*n* = 38) of DBN patients were diagnosed with episodic vestibular syndrome (EVS), 22.2% (*n* = 12) with chronic vestibular syndrome (CVS), and 7.4% (*n* = 4) with acute vestibular syndrome (AVS). Among all the patients, 51.9% of DBN patients had clear etiology, with central lesions of 29.6% and peripheral diseases of 22.2%. The most common diseases in DBN patients were cerebellar lesions (13.0%, *n* = 7) and vestibular migraine (13.0%, *n* = 7), followed by benign positional paroxysmal vertigo (7.4%, *n* = 4) and drug-related dizziness/vertigo (5.6%, *n* = 3). The other 48.1% of the patients had unknown etiology. 53.8% (14/26) of patients with idiopathic DBN had decreased semicircular canal function, with 42.9% (6/14) decreased posterior semicircular canal function. The posterior semicircular canal gain in DBN patients decreased compared to the anterior semicircular canal in the same conjugate plane. Patients with peripheral DBN were more prone to horizontal/torsional nystagmus during positional testing.

**Conclusion:**

In our study, DBN patients have a relative decrease in posterior semicircular canal gain, which is possibly a particular result found in a subset of downbeat nystagmus patients. The changes in nystagmus during positional testing may be helpful in distinguishing between peripheral and central causes.

## Introduction

Downbeat nystagmus (DBN) is one of the vertical nystagmus, which is characterized by a pathologic upward drift of gaze followed by a corrective downward saccade (1, 2). According to previous literature, the most common symptoms are unsteadiness of gait and to-and-fro vertigo (3). Moreover, patients frequently report blurred vision or oscillopsia that increases on lateral gaze. DBN is often associated with other oculomotor disorders, predominantly smooth pursuit deficits and impairment of the optokinetic reflex and visual fixation suppression of the vestibulo-ocular reflex (VOR). The etiological diagnosis of DBN has always been a clinical difficulty. Previous reports have shown that DBN is mostly caused by cerebellar and brainstem lesions, and can also be seen in some patients with peripheral vestibular diseases. Its causes include Arnold Chiari malformation (ACM), cerebellar degeneration, stroke, lithium or drug poisoning, Meniere’s disease, and vestibular neuritis (3–7). However, there is still a large proportion of DBN patients with unknown etiology, known as idiopathic DBN, with a proportion ranging from 25 to 65% (3, 8, 9). The pathophysiological basis of DBN still needs to be clarified.

According to previous literature, very few studies have reported the location, diagnosis and possible etiology and mechanism of downbeat nystagmus in China, which were almost case reports. Yang et al. presented a case of spontaneous, consistent DBN in the absence of vertiginous experience and the patient was completely relieved of the symptoms after surgical removal of the tumor, which was histologically confirmed as Grade I Ganglioglioma (10). Another report from two patients who developed reversible downbeat nystagmus while using antiepileptic drugs within the therapeutic range (11). In this study, more population of the medical history and examination data of patients with spontaneous DBN were retrospectively analyzed, in order to explore the location and diagnosis of DBN as well as the possible etiology and mechanism, and to provide the basis for the etiological diagnosis of dizziness/vertigo patients with DBN.

## Methods

A total of 3,471 patients admitted to vertigo center of our hospital from January 2018 to March 2021 were screened, and 54 patients were diagnosed with dizziness/vertigo with DBN by video-nystagmography (VNG). Detailed records of the included patient history, especially the following parameters related to the diagnosis and differential diagnosis of the disease.

dizziness/vertigo/gait instability: course of disease, onset form, duration, frequency of onset, triggering/alleviating factors, related eye symptoms (including oscillation, diplopia, blurred vision or blindness), related ear symptoms (including tinnitus, hearing loss, ear fullness), related cerebellar symptoms (ataxia, dysarthria), and other concurrent symptoms such as headache, fear of sound, photophobia, nausea, and vomiting.

Systematic inquiry on past history and family history, especially migraine, cardiovascular risk factors (such as hypertension, diabetes, hyperlipidemia), carsickness history, and other neurological, ophthalmological, otorhinolaryngological and medical diseases.

**Figure 1:**
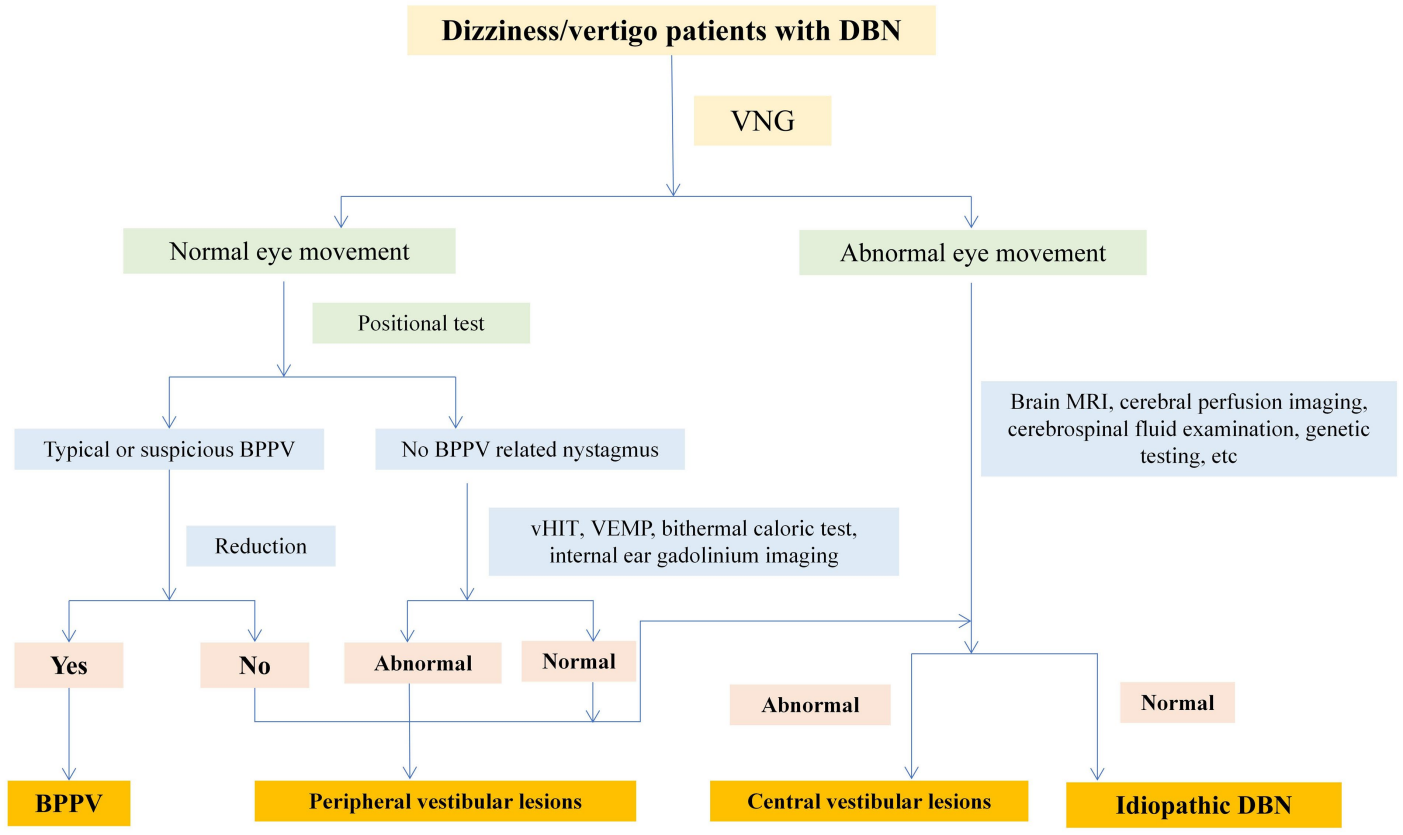
Diagnostic flow chart.

VNG was used to examine spontaneous nystagmus, positional nystagmus, smooth pursuit, saccades, and optokinetic nystagmus. Some patients underwent head-shaking nystagmus (HSN), vHIT, VEMP, head MRI, inner ear 3D-FLAIR MRI, and serum immunology examinations. One patient underwent brain CT perfusion scanning. Two experienced neurologists specializing in dizziness analyzed the above examination results, combined with patient medical history data, to conduct localization, qualitative analysis, and etiological diagnosis. Senior doctors (PG and XY) were in charged of the diagnoses based on the patient’s symptoms, signs and auxiliary examination results. Vestibular diseases were diagnosed according to the International Classification of Vestibular Diseases (ICVD) criteria, for instance, vestibular neuritis, vestibular migraine (VM) or meniere disease (MD). The diagnostic flow of central and peripheral diseases was shown in Figure 1.

### Video-nystagmography

Spontaneous nystagmus and positional nystagmus of all patients were recorded using VNG (Interacoustics, Middelfart, Denmark). Among them, 42 patients underwent head-shaking nystagmus with passive head shaking method and 50 underwent bithermal caloric testing. A canal paresis (CP) value of >25% indicates reduced unilateral horizontal semi-circular canals (SCC) function. A sum of the slow-phase velocity (SPV) values obtained by stimulating both ears with cold and hot water of the bilateral SCCs of ≤12°/s suggests reduced bilateral horizontal SCC function.

### video-head-impulse test

vHIT was performed using EyeSeeCam (Interacoustics, Middelfart, Denmark) to assess vestibulo-ocular reflex (VOR). vHIT evaluates VOR by testing all three pairs of SCCs, and calculates the average gain value and the asymmetry ratio of the two semicircular canals in the conjugate plane for each semicircular canal. Reduced SCC function is defined as a horizontal SCC gain less than 0.8 and a vertical SCC gain less than 0.7.

### vestibular-evoked myogenic potentials

oVEMP and cVEMP were detected by an evoked response auditory stimulator (Interacoustics, Middelfart, Denmark). Bilateral amplitude asymmetry ratio is normally defined as cVEMP≤0.35 and oVEMP≤0.33. The normal values of VEMP latency defined by our laboratory were cVEMP N18P25 (P1 16.90 ± 3.37 ms; N1 25.24 ± 4.07 ms) and oVEMP N13P18 (P1 15.9 ± 3.02 ms; N1 12.8 ± 2.47 ms). Failure to extract the waveform, asymmetry of amplitude beyond the normal range, or prolonged latency are all defined as abnormal results.

### Head MRI

Head MRI was performed on 30 patients with DBN using 3.0 T magnetic resonance imaging (siemens AmiraMR). T1 weighted image (T1WI), T2 weighted image (T2WI), Flair sequence and DWI sequence were improved.

### 3D-Flair MRI

Twenty-three DBN patients underwent 3D-flair labyrinthine Enhanced MRI under 3.0 T NMR apparatus. A 2-dose gadolinium contrast technique was used to perform secondary scans at 4-h intervals, including T1-weighted images (T1WI), T2-weighted images (T2WI), 3D-FLAIR sequence, delated-enhanced 3D-FLAIR sequence, and T2-SPACE sequences.

### Serum immunological indicators

Serum immunological tests were including blood routine, rheumatoid factor, C-reactive protein, antinuclear antibody, thyroid peroxidase antibody, thyroid globulin antibody.

### Statistical analysis

The data were collected through Excel and analyzed using the SPSS 26.0 software package. The measurement data were represented by mean ± standard deviation (SD). The counting data were expressed in frequency (percentage), and Fisher’s exact test was used for inter-group comparison. Student’s t-test was used for inter-group comparison of measurement data. Bilateral test was used for all data, and *p* < 0.05 was considered statistically significant.

## Results

### Demographic characteristic of patients

A total of 54 patients with dizziness/vertigo and DBN were included, accounting for 1.6% (54/3471) of patients with dizziness/vertigo during the same period. The mean age of included patients with DBN dizziness/vertigo was 59.3 ± 18.8 years (range 11–87 years; Table 1). The mean age of patients with central DBN was 47.5 ± 19.2 years (range 11–75 years) and that of patients with peripheral DBN was 55.7 ± 18.1 years (range 26–84 years), displayed as Table 2. The male to female ratio was 9:7 in the central DBN group and 4:8 in the peripheral DBN group. The age of peripheral DBN patients was older than that of central DBN patients, and females accounted for a slightly higher proportion, but there was no statistically significant difference between the two groups in gender and age.

**Table 1:** Demographic, clinical characteristics and laboratory findings of patients.

Variables	Value, *n* = 54
Age(years), mean ± SD	59.28 ± 18.81
Range	11–87
Male, n(%)	26(48.1)
Symptoms, n(%)	
Spontaneous or induced dizziness	30(55.6)
Vertigo	25(46.3)
Gait instability	18(33.3)
Oscillopsia	3(5.6)
Tinnitus	9(16.7)
Hearing change	8(14.8)
Vestibular syndrome, n(%)	
Episodic vestibular syndrome, EVS	38(70.4)
Chronic vestibular syndrome,CVS	12(22.2)
Acute vestibular syndrome, AVS	4(7.4)
Nystagmus intensity(°), mean ± sd	6.74 ± 3.98
Unilateral vestibular hypofunction, n1/n2(%)	17/50(31.5)
vHIT abnormal, n1/n2(%)	27/45(60.0)
cVEMP abnormal, n1/n2(%)	10/36(27.8)
oVEMP abnormal, n1/n2(%)	13/36(36.1)
MRI abnormal, n1/n2(%)	6/37(16.2)
3D-flair labyrinthine Enhanced MRI abnormal, n1/n2(%)	5/23(21.7)
Serological abnormalities, n1/n2(%)	11/36(30.6)

n1 represented the number of abnormal patients; n2 represented the number of patients examined.

**Table 2:** Comparison of peripheral and central patient characteristics and laboratory results.

Variables	Peripheral, *n* = 12	Central, *n* = 16	Statistics, Τ	*p*
Age(years), mean ± sd	55.7 ± 18.1	47.5 ± 19.2	1.146	0.264
Range	26–84	1–75		
Male, n(%)	4(33.3)	9(56.3)	–	0.276
Dizziness, n(%)	4(33.3)	12(75.0)	–	0.053
Unstable symptoms, n(%)	3(25.0)	6(37.5)	–	0.687
Induced nystagmus, n(%)	5(41.7)	2(12.5)	–	0.103
Nystagmus intensity, mean ± sd	6.50 ± 2.75	7.13 ± 4.52	0.426	0.674
Positional nystagmus test, n(%)	11(91.7)	8(50.0)	**–**	0.039
Unilateral vestibular hypofunction, n(%)	3(25.0)	6(37.5)	–	0.687
vHIT abnormal, n(%)	8 (72.7)#	7 (43.8)	–	0.239
cVEMP abnormal*, n(%)	3(33.3)	2(15.4)	–	0.609
oVEMP abnormal*, n(%)	5(55.6)	2 (15.4)	–	0.074
Serological abnormalities, n(%)	3(25.0)	3(18.8)	–	>0.999

*Peripheral and central DBN patients were tested in 9 and 13 cases, respectively. #Test was performed in 11 cases.

### Clinical manifestations

The main manifestations were spontaneous or induced dizziness, vertigo, and gait instability. Some patients complained of oscillopsia, tinnitus, and hearing changes (Table 1). The proportion of dizziness symptoms in peripheral DBN patients was 33.3% (*n* = 4) and that in central DBN patients was 75% (*n* = 12). Peripheral and central DBN patients with vertigo accounted for 50% (*n* = 6) and 37.5% (*n* = 6), respectively. However, the incidence of instability symptoms in both cases was 25% (*n* = 3) and 37.5% (*n* = 6). In addition, in peripheral DBN, 41.7% (*n* = 5) of patients had induced nystagmus; In central DBN, only 12.5% (*n* = 2) of patients have induced nystagmus. Therefore, dizziness is more commonly used as the chief complaint in central DBN, with a higher proportion of spontaneous attacks, while peripheral DBN patients are often accompanied by complaints induced or aggravated by changes in head and body position, but there is no significance between the two groups (Table 2).

### Features of DBN

The intensity of nystagmus in all DBN patients was 6.74 ± 3.98°/s, including 6.50 ± 2.75°/s for peripheral DBN and 7.13 ± 4.52°/s for central DBN. There was no significant difference between two groups, and the change of body position or shaking head stimulation had little effect on the SPV value of nystagmus in the 28 diagnosed DBN patients (*p* > 0.05). However, a separate analysis of 7 patients diagnosed with VM showed that the intensity of nystagmus in these patients decreased in the overhanging head position (*p* < 0.05). In addition to the intensity of the nystagmus, 11 of the 12 patients with confirmed peripheral DBN had horizontal and/or torsional nystagmus at the time of the location test, while only 8 of the 16 patients with confirmed central DBN had horizontal and/or torsional nystagmus during the location test (Tables 1, 2).

#### Bithermal caloric testing

Bithermal caloric testing were performed in 50 of the 54 patients. Finally, 17 cases (31.5%) met the diagnosis of unilateral decreased vestibular function, including 3 cases of peripheral DBN and 6 cases of central DBN, and there was no statistical difference in incidence between the two groups (*p* > 0.05).

#### vHIT

**Figure 2:**
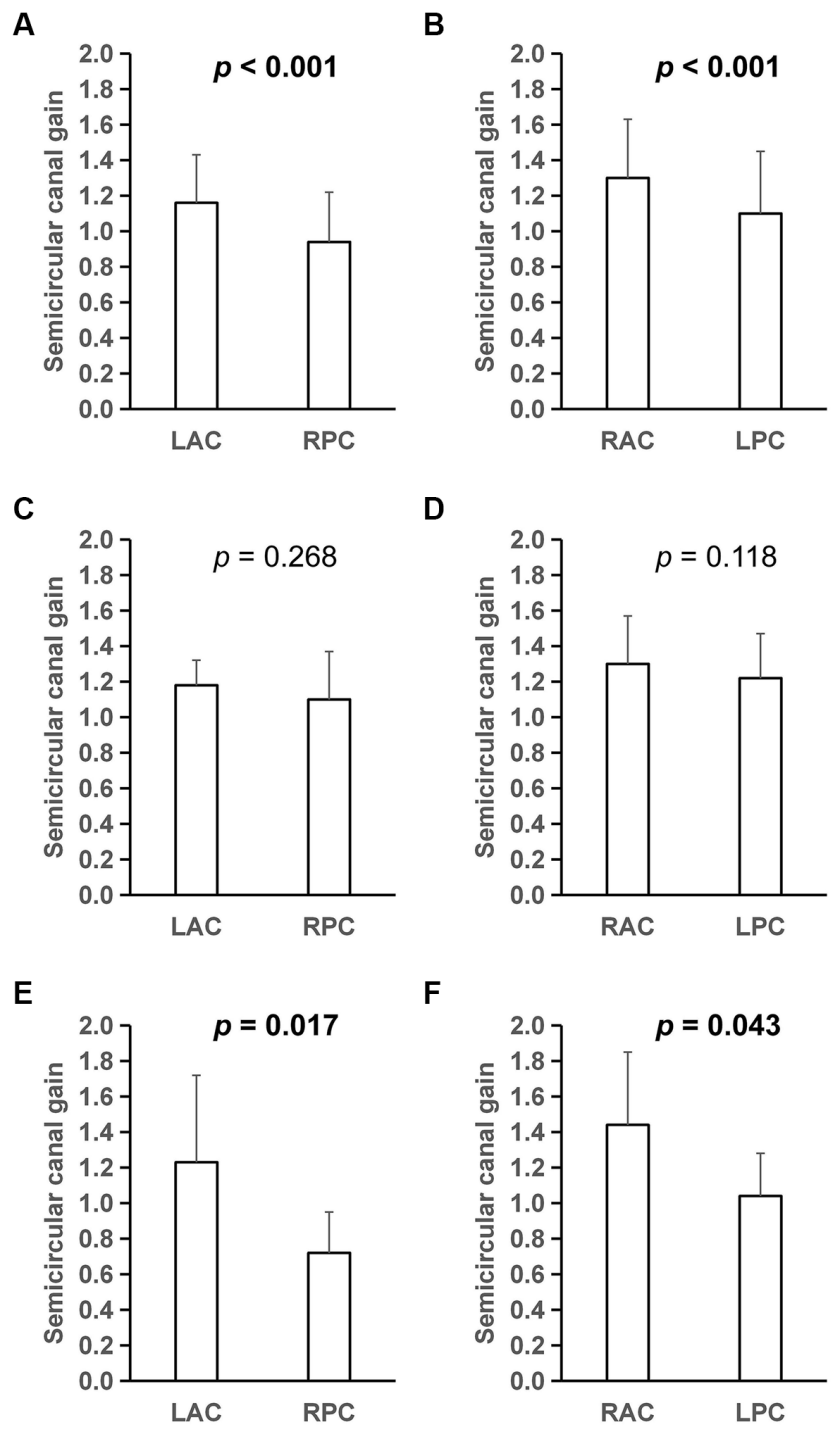
Semicircular canal gain detected by vHIT. **(A,B)** were test results of 45 patients, **(C,D)** were results of 7 VM patients, and **(E,F)** were results of 7 patients with cerebellar-related diseases. vHIT, video-head-impulse test; VM, vestibular migraine; LAC, Light anterior semi-circular canal; LPC, Light posterior semi-circular canal; RAC, Right anterior semi-circular canal; RPC, Right posterior semi-circular canal.

Of the 45 DBN patients who underwent vHIT, 60.0% (*n* = 27) had abnormalities manifested as reduced SCC gain, recessive/dominant saccade wave, or reduced SCC gain combined with saccade wave. Among the 27 cases of abnormal vHIT, 48.1% (13/27) showed decreased SCC gain, most of which were posterior SCC dysfunction (84.6%, *n* = 11), and 1 case was accompanied with decreased SCC gain. The posterior SCC gain in DBN patients was lower than that of anterior SCC in the conjugate planes, and the difference was statistically significant (*p* < 0.05). In peripheral DBN and central DBN, there was no significance in SCC gain and asymmetry ratio of two SCC gain (*p* > 0.05). However, no difference in vertical SCC gain was found in 7 VM patients (*p* > 0.05), while a relative decrease in posterior SCC gain was found in cerebellar related lesions (*p* < 0.05; Figure 2).

#### VEMP

In this study, 36 patients underwent VEMP examination. The overall abnormal rate of cVEMP was 27.8% (10/36), the peripheral abnormal rate of DBN was 33.3% (3/9), and the central was 15.4% (2/13), but the difference between groups was not statistically significant (*p* > 0.05). Moreover, the overall abnormality rate of oVEMP was 36.1% (13/36), with a clear peripheral DBN abnormality rate of 55.6% (5/9) and central DBN abnormality rate of 15.4% (2/13). The abnormal rate of oVEMP in peripheral group had a higher trend than that in central, but without significance (*p* > 0.05).

#### Head MRI

A total of 30 patients underwent head MRI examination. There were 2 cases of cerebellar atrophy, 1 case of cerebellar infarction, 1 case of cerebellar subtonsillar hernia, 1 case of cerebellar hemisphere infarction and 1 case of cavernous hemangioma in cerebellar hemisphere.

#### 3D-flair labyrinthine enhanced MRI

A total of 23 patients underwent 3D-flair MRI, 21.7% (*n* = 5) had abnormalities, including right petrous apex cholestatoma, left posterior inferior cerebellar artery adjacent to the left acoustic nerve REZ area, right internal auditory auditory auditory neuroma, basilar artery tortuosity, and right hydrolabyrinthia.

#### Serum immunological indicators

The levels of serum thyroglobulin antibody, thyroid peroxidase antibody, C-reactive protein, rheumatoid factor, antinuclear antibody and antiphospholipid antibody in 36 DBN patients were detected. 30.6% (*n* = 11) of the patients had one or more indexes abnormal, including 3 cases of peripheral DBN and 3 cases of central DBN, and there was no statistical difference (*p* > 0.05).

#### Medication

Among DBN patients included in this study, 3 cases were considered to be drug-related, respectively, quetiapine fumarate, estazolam and trastuzumab, and their dizziness symptoms began to appear after medication, mainly presented as persistent dizziness, without vertigo and unsteady walking, and no tinnitus or hearing changes.

### Etiological classification

According to the pattern of dizziness/vertigo symptoms, 70.4% (*n* = 38) were diagnosed as episodic vestibular syndrome, 22.2% (*n* = 12) as chronic vestibular syndrome, and 7.4% (*n* = 4) as acute vestibular syndrome.

51.9% (*n* = 28) of DBN patients have a clear etiology, the diagnostic flow chart was shown in Figure 1. Among them, central lesions accounted for 29.6% (*n* = 16), and peripheral lesions accounted for 22.2% (*n* = 12). The most common diseases are cerebellar lesions (13.0%, *n* = 7) and vestibular migraine (13.0%, *n* = 7), followed by benign positional paroxysmal vertigo (7.4%, *n* = 4) and drug related DBN (5.6%, *n* = 3). Among the central DBN, 7 patients met the diagnostic criteria for vestibular migraine and probable vestibular migraine jointly developed by the Barany Society’s Vestibular Disorders Classification Committee and the International Headache Society (IHS) Migraine Classification Subcommittee. Among the 7 patients diagnosed with cerebellar lesions, 2 were confirmed by genetic testing as paroxysmal ataxia type 2 (EA2). Another 48.1% (*n* = 26) of patients have an unknown etiology, which is idiopathic DBN. 53.8% (*n* = 14) of patients with idiopathic DBN have decreased SCC function, with 42.9% (6/14) having decreased posterior SCC function (Table 3).

**Table 3:** Patient etiology diagnosis.

Etiological diagnosis	Cases, n(%)
Central etiology	16(29.6)
Cerebellar lesions	7(43.8)
*Episodic ataxia type 2*	*2(28.6)*
*Cerebella tonsil herniation*	*1(14.3)*
*Viral cerebellitis*	*1(14.3)*
*Cerebellar hemisphere perfusion*	*1(14.3)*
*Cerebellar hemisphere infarction*	*1(14.3)*
*Cerebellar hemisphere cavernous malformations*	*1(14.3)*
Vestibular migraine,VM	7(43.8)
*Confirmed VM*	*2(28.6)*
*Possible VM*	*5(71.4)*
Drug related	2(12.5)
Q*uetiapine fumarate*	*1(50.0)*
*Estazolam*	*1(50.0)*
Peripheral etiology	12(22.2)
BPPV	4(33.3)
*Canalolithiasis of the posterior canal pc-BPPV*	*3(75.0)*
*Canalolithiasis of the anterior canal ac-BPPV*	*1(25.0)*
Light cupula	1(8.3)
Cholesteatoma	1(8.3)
Acoustic neuroma	1(8.3)
Vestibular paroxysmia, VP	1(8.3)
Meniere’s disease	2(16.6)
Hunt syndrome	1(8.3)
Drug related (trastuzumab)	1(8.3)
Etiology unknown	26 (48.1)
Semicircular canals function abnormal	14(53.8)
P*osterior semicircular canal dysfunction*	*6(42.9)*
*Horizontal semicircular canal dysfunction*	*8(57.1)*
Without semicircular canal dysfunction	12(46.2)

## Discussion

The concept of DBN was first described by Dr. David in the mid-1960s. Theoretically, DBN caused by reduced posterior SCC function or superior SCC hyperfunction are often accompanied by torsion components, and only when bilateral SCC involvement is basically symmetric can pure sDBN be produced (6). Other scholars believe that the vertical SCC is smaller than the horizontal SCC when it merges the feet before entering the vestibule, and has greater additional resistance to volume expansion. In terms of this feature, the endolymph flow obstruction caused by pressure gradient in the vertical SCC will be smaller than that in the horizontal SCC (12). Vertical nystagmus is relatively rare in clinical practice for all of the above reasons, which is why DBN was previously thought to be associated with structural abnormalities or dysfunction of central vestibular structure, especially cerebellar lesions.

With the continuous development of relevant studies, it has been proved that brainstem and peripheral vestibular diseases could also cause DBN (4, 5, 13–15). Common etiological diagnoses include cerebellar degenerative diseases, stroke, vestibular migraine, Chiari malformation, spinocerebellar ataxia type 6, parataxia type 2, Meniere’s disease, sub-vestibular neuritis, etc. However, there are still some cases with unclear etiology, the proportion of which ranges from 25 to 65%. Hypotheses related to the occurrence of DBN include the asymmetry of the vertical vestibular ocular reflex (VOR), the inherent asymmetry of the central vestibular ocular movement system in the vertical direction, the up VOR pathway disinhibition, the disturbance of the vertical stationary tracking system, and the impairment of the vertical ocular movement neural integration function, etc. It may involve pathophysiological mechanisms such as gene susceptibility, abnormal ion channels and changes in neurotransmitter levels (16–20). In this study, 48.1% of patients with unknown etiology were diagnosed with idiopathic DBN, similar to previously reported cases. Among sDBN with clear etiology, there are both central and peripheral diseases, such as vestibular migraine, cerebellar lesions, BPPV and light cupula. It is worth emphasizing that, the mechanism of light cupula remains to be clarified. However, theories of decreased specific gravity of cupula, changes in surrounding endolymph specific gravity, light otolith or morphological changes of cupula are taken for the causes of peripheral lesions, so our study temporarily attributed light cupula to peripheral lesions (21).

### DBN with clear etiology

Among the 28 patients with DBN who were clearly diagnosed, the central causes were cerebellar lesions, VM, and drugs, and peripheral diseases were MD, cholesteroma, drugs, acoustic neuroma, Hunt syndrome, light cupula and VP. According to previous studies, the cerebellum has an inhibitory effect on the upward eye movement pathway, and when the cerebellum is involved, it can lead to the loss of inhibition of the upward pathway, resulting in DBN (6, 22). However, VM is currently considered to be a functional disease, but the mechanism is not clear. It is generally considered to be a subtype of migraine, with variable types of nystagmus. Besides, we found that the DBN intensity of 7 VM patients decreased in the suspended head position, which may be a characteristic manifestation of nystagmus.

In this study, two patients developed DBN after taking quetiapine fumarate and estazolam respectively, which may be related to their effects on brain transmitter levels or effects. Currently, the reported drugs that can cause DBN include lithium, antiepileptic drugs, ranitidine, pregabalin, etc., which are mostly believed to be related to the effect of drugs on the secretion of neurotransmitters (histamine, γaminobutyric acid) in the nervous system and the reduction of excitatory input of the vestibular nucleus (especially superior vestibular nucleus) in the brain stem (19, 23, 24). However, no cases of DBN induced by quetiapine fumarate have been reported. Miguel Paciuc-Beja et al. (25) reported two cases of DBN in patients using antidepressants (5-HT reuptake inhibitors) under anesthesia, which was believed to be related to the effect of opioid combined antidepressants on cerebellar function. Quetiapine fumarate has antagonistic effect on both histamine H1 receptor and 5-HT. Hence, it is theoretically possible to affect cerebellar excitability of brainstem and induce DBN production by regulating histamine and 5-HT levels. But the exact mechanism needs further study.

For peripheral DBN, the occurrence of nystagmus is considered to be related to the combined effects of peripheral vestibular organ involvement, such as the involvement of multiple SCCs and/or otolith organs in MD patients due to non-selective hydrops, and the destruction of multiple SCCs and/or vestibular nerves by cholestatoma. DBN occur when the peripheral involved structure predominates in affecting upward eye movement, which is rare but not impossible. In this study, although DBN was present in patients with peripheral lesions, there were more horizontal and/or torsional nystagmus than in the position testing, which was considered to be related to damage of peripheral VOR pathway. What is more, according to Ewald’s first law, in the case of peripheral vestibular disease, due to the corresponding relationship between the semicircular canal and the extraocular muscle, when the vertical semicircular canals are involved, the combined force of the superior and inferior oblique and rectus muscles could cause the vertical movement of the eye, meanwhile, the horizontal and/or torsion movements are also involved (26). However, due to the functional limitations of the instrument used, our study could not quantify the difference between torsional nystagmus in peripheral and central lesions.

### DBN with unknown etiology

In this study, the etiology of 26 patients with DBN was not clear, among which 14 had decreased SCC function and 6 had posterior SCC dysfunction. However, due to the lack of eye movement index, otolith pathway evaluation, cerebral perfusion imaging (arterial spin labeling, ASL), SVV, nystagmus time constant and other indicators conducive to the evaluation of central differentiation, it is still unable to be clearly located and qualitative diagnosis.

### vHIT and VEMP

vHIT can separately assess the function of six SCCs at high frequency, and its gain reduction is more considered as strong evidence for peripheral lesions, while the conclusions for central lesions are not clear. The posterior SCC gain in DBN patients included in this study was lower than that of anterior in the conjugate plane. This result is consistent with the pathophysiological changes that cause DBN, that is, posterior semicircular VOR disorder directly leads to weakened signals in the superior oblique and inferior rectus pathways of vestibular, resulting in slow upward movement of eyeballs, and then causing DBN. Therefore, it is speculated that DBN patients may have a relative decrease in posterior SCC function. However, analysis of patients diagnosed with VM and cerebellar lesions found different results, suggesting that cerebellar lesions may present peripheral-like manifestations, and that sDBN in VM patients may be more related to the vestibular cortical center. Since the bithermal caloric testing mainly evaluated the low frequency function of the horizontal SCC, no strong evidence has been provided in terms of the pathogenesis of DBN.

VEMP is mainly used to evaluate the otolith-organ pathway. In 2015, Bremova et al. (27) also found abnormal oVEMP in their study on VEMP in DBN patients, but the included population were only with central lesions. The author believed that the abnormal oVEMP was caused by the damage of cerebellar related vertical eye movement pathway, which resulted in enhanced otolith-ocular pathways signal. In this study, the abnormal rate of oVEMP in peripheral DBN patients was higher than that in central DBN, but without significantce, and it cannot be ruled out that it is related to the small sample size.

Our study carefully analyzed the clinical symptoms and examination indicators of patients with downbeat nystagmus. As a retrospective study in nature, it might have the following shortcomings: the number of patients was small, and the examination results of some patients were not comprehensive enough, except for 2 EA2 patients with a family history of dizziness and suspected ataxia, the remaining 52 patients did not provide a clear family history of dizziness or ataxia-related diseases, due to no genetic tests were performed, so the possibility of hereditary diseases in some patients cannot be ruled out. Hence, the applicability of the conclusion needs to be further verified by a larger population. Moreover, differences in torsional nystagmus between peripheral and central lesions could not be quantified on account of functional limitations of instruments.

## Conclusion

This study suggests that DBN patients have a relative decrease in posterior semicircular function, but the differential diagnostic significance of peripheral and central lesions is still unclear. In addition, the presence of horizontal and/or torsional nystagmus in positional tests may be more supportive of peripheral diagnosis. Although the exact mechanism and conduction pathway of DBN are still controversial, its accurate diagnosis remains a clinical challenge. However, with the continuous development of detection technology, combined with detailed medical history, physical examination, follow-up observation, and even experimental treatments, we believe that the diagnostic rate of such patients would continue to improve.

## Data availability statement

The original contributions presented in the study are included in the article/supplementary material, further inquiries can be directed to the corresponding authors.

## Ethics statement

The studies involving humans were approved by The First Hospital of Hebei Medical University. The studies were conducted in accordance with the local legislation and institutional requirements. The participants provided their written informed consent to participate in this study.

## Author contributions

SZ: Conceptualization, Data curation, Formal analysis, Investigation, Methodology, Software, Writing – original draft. YL: Conceptualization, Formal analysis, Investigation, Methodology, Writing – original draft. WW: Conceptualization, Investigation, Methodology, Project administration, Writing – original draft. YW: Investigation, Methodology, Project administration, Writing – original draft. SY: Investigation, Methodology, Project administration, Writing – original draft. TZ: Investigation, Methodology, Project administration, Writing – original draft. DL: Investigation, Methodology, Project administration, Writing – original draft. SL: Methodology, Project administration, Writing – original draft. YH: Methodology, Project administration, Writing – original draft. XY: Conceptualization, Supervision, Validation, Writing – review & editing. PG: Conceptualization, Investigation, Supervision, Validation, Writing – review & editing.
